# The role of ATP‐binding cassette subfamily G member 1 in tumor progression

**DOI:** 10.1002/cam4.7285

**Published:** 2024-06-19

**Authors:** Xu Xinyi, Yang Gong

**Affiliations:** ^1^ Central Laboratory, The Fifth People's Hospital of Shanghai Fudan University Shanghai China; ^2^ Cancer Institute Fudan University Shanghai Cancer Center Shanghai China; ^3^ Department of Oncology Fudan University Shanghai Medical School Shanghai China

**Keywords:** ABCG1, chemoresistance, stemness, tumor microenvironment, tumor progression

## Abstract

**Background:**

ATP‐binding cassette subfamily G member 1 is mostly known as a transporter for intracellular cholesterol efflux, and a number of studies indicate that ABCG1 also functions actively in tumor initiation and progression. This review aimed to provide an overall review of how ABCG1 acts in tumor progression.

**Method:**

A comprehensive searching about ABCG1 and tumor was conducted up to November 2023 using proper keywords through databases including PubMed and Web of Science.

**Result:**

Overall, ABCG1 plays a crucial role in the development of multiple tumorigenesis. ABCG1 enhances tumor‐promoting ability through conferring stem‐like properties to cancer cells and mediates chemoresistance in multiple cancers. Additionally, ABCG1 may act as a kinase to phosphorylate downstream molecules and induces tumor growth. In tumor microenvironment, ABCG1 plays a substantial role in immunity response through macrophages to create a tumor‐favoring circumstance.

**Conclusion:**

High expression of ABCG1 is usually associated with poor prognosis, which means ABCG1 may be a potential biomarker for early diagnosis and prognosis of various cancers. ABCG1‐targeted therapy may provide a novel treatment for cancer patients.

## Graphical abstract

ABCG1 plays a critical role in promoting tumor development mainly through four aspects including tumor initiation and progression, chemoresistance, cancer cell stemness and tumor microenvironment. High expression of ABCG1 in multiple cancers is usually associated with a poor prognosis.

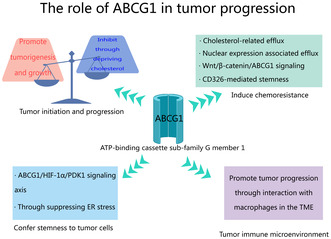

## 1 INTRODUCTION

The ATP‐binding cassette (ABC) transporter superfamily is expressed ubiquitously in prokaryotes and eukaryotes to transport substrates including peptides, amino acids, sugars, metabolites, drugs, and other massive hydrophobic compounds across the cell membrane.^1^ Up to now in human, 48 ABC superfamily members have been identified and can be further classified into 7 subfamily from ABCA to ABCG.^2^ Among them, ATP‐binding cassette subfamily G is a type of half‐sized transporter in human including five main members named ABCG1, ABCG2, ABCG4, ABCG5, and ABCG8.^3^ Most ABCG members are associated with sterol transportation except ABCG2 which is identified to be more relevant to drug resistance and cancer therapy.^4^

ABCG1, one of the ABCG members, is detected predominantly in intracellular endosomes in various tissues and the detailed function of ABCG1 is cell type‐dependent.^5^ It is widely reported that ABCG1 plays a substantial role in stabilizing intracellular sterol homeostasis and distribution by controlling the efflux of cholesterol from cells to HDL.^6^ Abnormal expression of ABCG1 affecting lipid metabolism and is involved in pathogenesis and progression of diverse diseases such as atherosclerosis, Alzheimer's disease, impairment in glucose tolerant and diabetes.^7^, ^8^, ^9^ Especially in atherosclerosis, the role of ABCG1 is demonstrated to be complex. Lacking of ABCG1 in mice increases early atherosclerosis lesions but delays the progression of advanced atherosclerotic lesions.^7^

So far, ABCG1 has been confirmed to be involved in cholesterol efflux and links to some metabolic diseases, yet less is mentioned about the relationship between ABCG1 and cancer growth. There is still a large gap in the systematic elaboration of how ABCG1 exactly acts within tumors. Actually, ABCG1 is also critical in tumor progression. Tian et al. reported that ABCG1 may work as an oncogene in lung cancer, modulating cancer cells proliferation, migration, invasion, and apoptosis.^10^ In fact, ABCG1 induces not only the cholesterol but also some antitumor drugs efflux from cells and enhance chemoresistance to prevent cells from death. ABCG1 can also confer stemness to tumor cells, improving the survival advantage of cancer stem cells. In addition to tumor cells, ABCG1 is identified to interact with cells in the tumor microenvironment. ABCG1 may potentially shift the immunity response to a tumor‐favoring condition through macrophages to promote tumor growth. Based on different activities of ABCG1 in tumors, it can be a potential biomarker for early diagnosis, chemoresistance progression, and prognosis of multiple cancers.^11^, ^12^, ^13^, ^14^, ^15^, ^16^ Figuring out the detailed role and the underlying mechanisms of ABCG1 within tumors may provide a promising target for precise therapy especially for ABCG1‐overexpression tumor patients. The purpose of this review is to provide an in‐depth review of the tumor‐associated aspect of ABCG1 in tumor progression.

## 2 ABCG1 AND REVERSE CHOLESTEROL TRANSPORT

**FIGURE 1:**
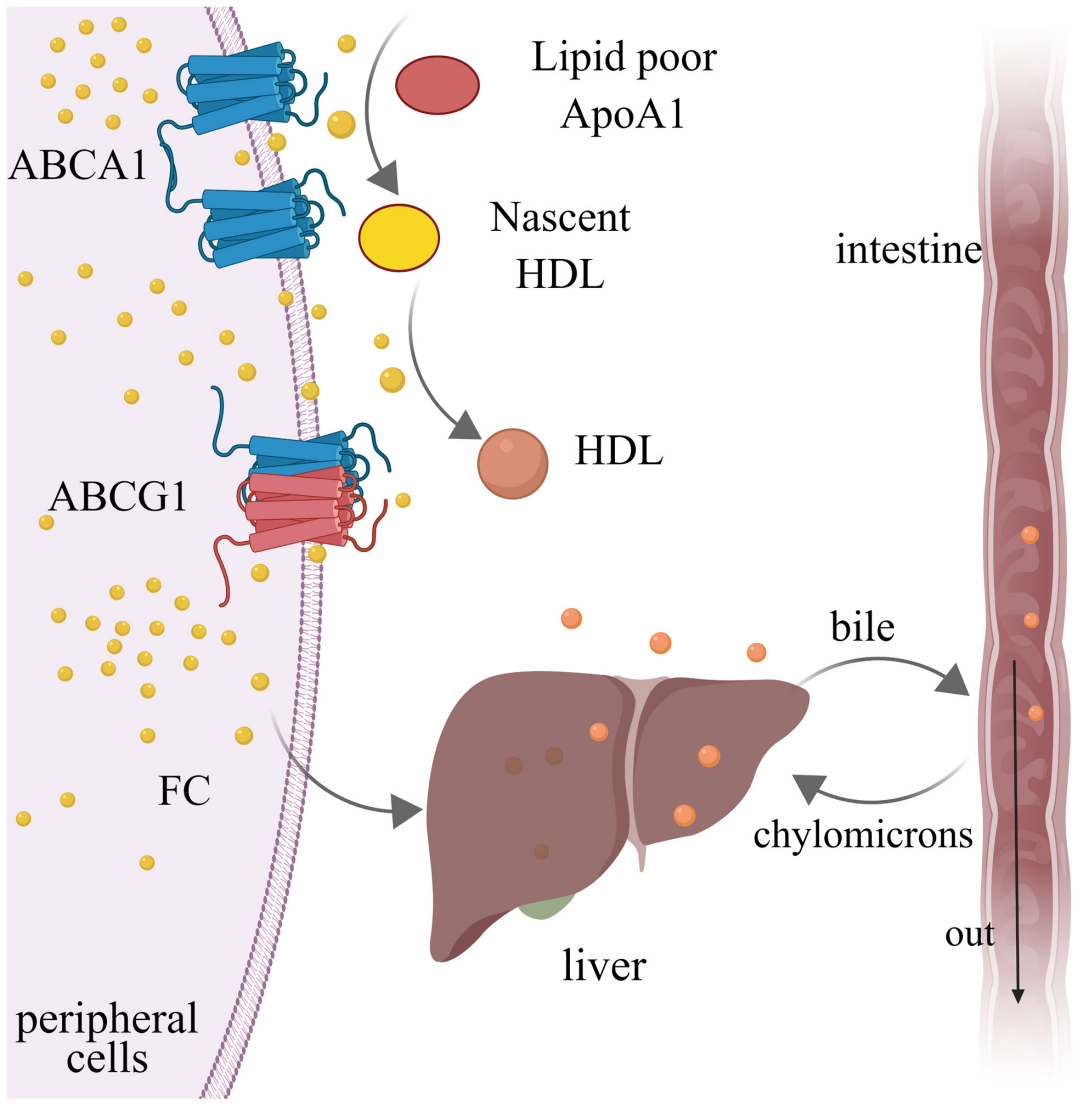
ABCG1 works together with ABCA1 and apoA1 in peripheral cells to transform free cholesterol (FC) into HDL and subsequently transports it to liver, which is termed as reverse cholesterol transport. Part cholesterol is secreted to intestine with bile after degrading by the liver and some of them will be reabsorbed and recycle in the plasma as chylomicrons, while the remaining will be secreted out.^18^

As a half‐sized transporter, ABCG1 consists of one membrane‐spanning domain (MSD) and one highly conserved nucleotide‐binding domain (NBD) at the N‐terminus, which forms a homodimer to functionally transport substrate across a membrane.^17^ Specifically, subfamily ABCA member ABCA1 works to transport free cholesterol (FC) from peripheral cells to lipid poor apoA1 to generate nascent HDL, which reversely acts as substrates to accept ABCG1‐mediated FC that is finally changed as mature HDL in liver and moved along with biliary excretion^18^ (Figure 1). In macrophages, the upregulated expression of ABCG1 mediates cholesterol efflux and links HDL levels to atherosclerosis risk.^19^ Both ABCG1 and HDL are pivotal in reverse cholesterol transport (RCT) pathway to remove and excrete excessive cholesterol.

## 3 ABCG1 INHIBITS TUMOR PROGRESSION BY DEPRIVING CHOLESTEROL

**FIGURE 2:**
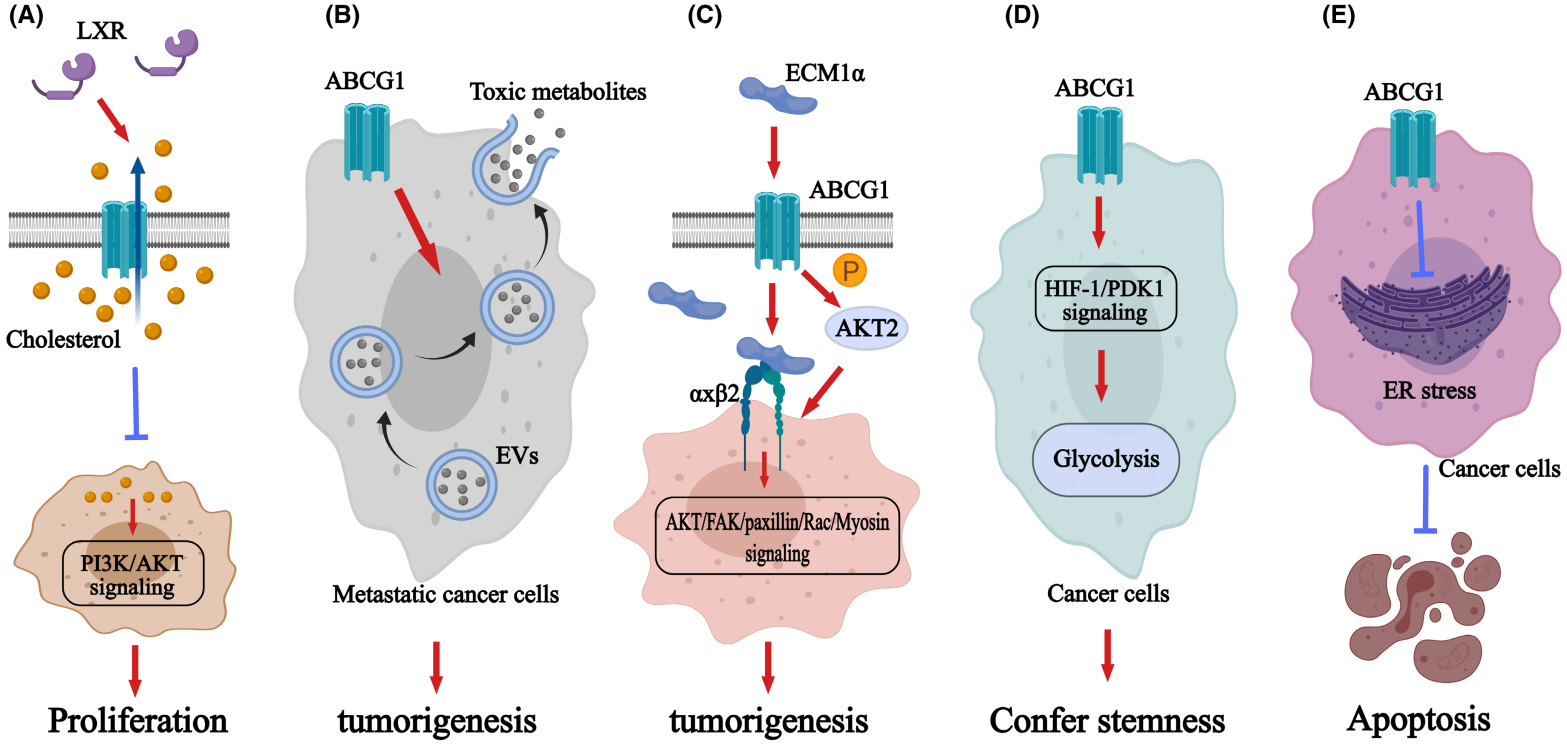
The role of ABCG1 in tumors. (A) ABCG1‐mediated cholesterol efflux in cancer cells can be induced by LXR to inhibit the phosphorylation of PI3K/Akt signaling.^24^ (B) ABCG1 mediates excessive intracellular cholesterol efflux via ERs to prevent cancer cells from cytotoxic lipid and promotes tumor growth.^32^, ^43^ (C) ECM1α induces the expression of ABCG1 to activate the Akt/FAK/paxillin/Rac/myosin signaling, which eventually promotes tumor development. ABCG1 can also act as a kinase to phosphorylate Akt directly.^26^ (D) The H19/HIF‐1/PDK1 signaling can be induced by ABCG1, which confers cancer cells stemness through increasing glycolysis.^30^, ^44^ (E) ABCG1 reduces cancer stem cell apoptosis by suppressing ER stress, and enhancing cell stemness.^34^, ^35^

The upregulated expression of ABCG1 is potential to kill cancer cells. One of the reasons may rely on the altered cholesterol levels in tumor cells, since ABCG1 plays a pivotal role in sterol transporting. Cholesterol is indispensable in cell membranes, and presents as critical energy and nutrient in many cellular physiological activities especially in oncogene‐transformed cancers. The elevated levels of cholesterol are required to sustain their rapid growth and proliferation.^20^ Besides, cholesterol is necessary to form cholesterol‐rich membrane rafts essential for cell viability and cell membrane integrity.^21^ Based on the fact that ABCG1 stabilizes sterol homeostasis through facilitating intracellular cholesterol efflux to HDL, upregulation of ABCG1 may deprive cholesterol for cellular survival, resulting in suppression of proliferation and increased apoptosis.^22^ ABCG1 is proved to inhibit the proliferation of hematopoietic stem and multipotential progenitor cells (HSPCs) via cholesterol efflux.^23^ In tumor cells, the ABCG1‐dependent cholesterol efflux also leads to an anti‐proliferation effect in breast cancer,^22^ prostate cancer,^24^ and clear cell renal cell carcinoma^25^ (Table 1). The underlying mechanism may lie on the decreased concentration of cholesterol in cell membrane lipid rafts that is mediated by ABCG1 to downregulate the phosphorylation of the PI3K/Akt signaling, resulting in apoptosis^24^ (Figure 2). Thus, the decrease of cellular cholesterol concentration induced by ABCG1 is associated with cancer cell death. Although ABCG1 exhibits an anti‐tumor effects from some tumors through excessive cholesterol efflux, the outcomes of clinical lung cancer patients treated with cholesterol depletion strategy are still dismal.^31^ There must exist some other offset mechanisms conferring tumor cells an adaptive ability.

**TABLE 1:** The detailed role and function of ABCG1 in tumor progression.

Type	Detailed role	Function way	References
Ovarian cancer	Promote tumorigenesis	Induces ECM1α‐αXβ2/AKT/FAK/paxillin/Rac/Myosin signaling	[26, 27]
		Acts like a kinase and directly phosphorylates AKT2	
	Induce chemoresistance	ECM1α‐αXβ2/CD326 signaling	
	Confer stemness		[26]
	Interact with TME		[28]
Breast cancer	Anti‐proliferation	Declined PI3K/Akt signaling phosphorylation induced by LXR‐ABCG1‐mediated cholesterol efflux	[22]
	Induce chemoresistance		[29]
	Confer stemness	H19/HIF‐1α/PDK1 signaling	[30]
Lung cancer	Promote tumorigenesis		[10]
	Confer stemness		
	Interact with TME	Induces pro‐tumorigenic role of TAM‐like macrophages through cholesterol efflux	[31]
Colon cancer	Promote tumorigenesis		[32]
	Induce chemoresistance		[33]
Glioma	Promote tumorigenesis		[34]
	Confer stemness	Suppression of ER stress	[35]
Prostate cancer	Anti‐proliferation	Declined PI3K/Akt signaling phosphorylation induced by LXR‐ABCG1‐mediated cholesterol efflux	[24]
Clear cell renal cell carcinoma	Anti‐proliferation	Declined PI3K/Akt signaling phosphorylation induced by LXR‐ABCG1‐mediated cholesterol efflux	[25]
Cholangiocarcinoma	Promote tumorigenesis		[36]
Lung adenocarcinoma	Induce chemoresistance		[37]
Acute myeloid leukemia	Induce chemoresistance		[14]
Pancreatic adenocarcinoma	Induce chemoresistance		[38]
Osteosarcoma	Induce chemoresistance	An independent drug efflux mechanism related to nuclear ABCG1 expression	[39]
Esophageal cancer	Induce chemoresistance		[40]
Adrenocortical carcinoma	Induce chemoresistance	Altered sterol storage	[41]
Hepatic cell carcinoma	Induce chemoresistance	Wnt/β‐catenin signaling	[42]

Abbreviations: TME, tumor microenvironment.

## 4 ABCG1 PROMOTES TUMORIGENESIS

### 4.1 Association of ABCG1 with extracellular vesicles and cancer cell growth

ABCG1 may prevent cancer cell from accumulation of cytotoxic lipids through extracellular vesicles. Extracellular vesicle is a kind of secreted structure consisting of lipid bilayer membranes, which can be classified into two types, ectosomes and exosomes.^45^ EVs, especially exosomes, are identified to remove excess and/or unnecessary substrates from cells, including kinds of lipids.^46^ Lipid metabolism affects many cellular processes and the balance between cell‐reliable and cell‐disrupted lipid.^47^ ABCG1 is detected to transfer sterol to the outer leaflet of the plasma membrane through endosome vesicle, which is essential for maintaining tissue lipid homeostasis.^5^, ^43^ Actually, the lipids efflux induced by ABCG1/EVs is also critical for the viability of cancer cells because the levels of both are largely increased and the depletion of ABCG1 leads to the accumulation of EVs containing lipids in cancer cells.^32^ With rapid growth, aggregation, and the Warburg effects of malignant tumor cells, the established acidic microenvironment results in more secretion of exosomes containing various metabolites, which further attenuates toxic accumulation.^32^, ^48^, ^49^, ^50^ In conclusion, ABCG1 plays a vital role in promoting cancer cell growth and tumor progression through lipids efflux via EVs. Both ABCG1 and EVs are important for tumorigenesis.

### 4.2 ABCG1 activates the AKT/FAK/paxillin/Rac/Myosin signaling to promote tumorigenesis

The expression of ABCG1 is significantly upregulated in variant cancers including metastatic colon cancer,^32^ cholangiocarcinoma,^36^ glioma,^34^ lung cancer,^10^ and ovarian cancer.^26^ ABCG1 functions actively in diverse tumors, and may facilitate cancer cell growth through mediating the phosphorylation of cellular signaling molecules. Some signal pathways are found to regulate various biological processes including tumor cell growth, proliferation, differentiation, apoptosis, immunity environment, and tumor chemotherapy.^51^, ^52^ Recently, we reported that the binding of extracellular matrix protein‐1α (ECM1α) to integrin αXβ2 activates the AKT/FAK/paxillin/Rac/Myosin signaling to promote ovarian tumorigenesis.^26^ ABCG1 acts as a midstream molecule induced by ECM1α, to promote the phosphorylation of AKT/FAK/paxillin/Rac/Myosin signaling and tumor growth. Since ABCG1 is detected to phosphorylate AKT, whether it also activates the other signal pathways to regulate more tumor types still need more investigations, but this kind of findings will shed a light on the novel function of ABCG1 in cancer development.

### 4.3 ABCG1 is a kinase?

Based on our research, ABCG1 acts as a kinase and directly phosphorylate downstream signals to promote ovarian tumorigenesis.^26^ Kinases are enzymes that transfer phosphate groups from high‐energy donor molecules like ATP/GTP to specific target substrates. Aberrant kinase signaling may result in malignant transformation and contribute to tumor development and progression.^53^ The identification of a key kinase will provide an ideal target to a successful molecular target therapy in cancer, which is becoming one of the most promising cancer therapy due to its high drug‐delivery specificity compared with conventional ones.^54^ Since we identified that ABCG1 can phosphorylate AKT2 in ovarian cancer, given the poor curative effects of ovarian cancer, the kinase role of ABCG1 may provide a therapeutic target for ovarian tumorigenesis after further investigations.

## 5 ABCG1 AND CHEMORESISTANCE

ABCG1 may confer cancer cell chemoresistance to promote tumor progression. Drug resistance is frequently developed during the clinical treatment, causing reduced responsiveness or tumor relapse, which has been a great obstacle to improve complete cure rate and overall survival of cancer patients.^55^ One of the mechanisms underlying may be the overexpression of several transmembrane transporters in tumor cells. The chemoresistance conferred in cells is distinct in different tumors caused by different ABC transporters.^56^ Among all ABC transporters, ABCB1 is well studied. Owing to broad substrate specificity, ABCB1 displays cross‐resistance to many different cytotoxic drugs, termed as multidrug resistance (MDR).^57^ And a study showed that ABCG1 may influence the chemoresistance function of ABCB1 through the altered cholesterol level in cells.^58^ Recently, the significant upregulation of ABCG1 is also detected to be directly associated with chemoresistance in multiple cancers.^14^, ^27^, ^29^, ^33^, ^37^, ^38^, ^39^, ^40^ Thus, ABCG1 may be another multidrug resistant molecule in tumor through active efflux of antitumor drugs from cancer cells.

**FIGURE 3:**
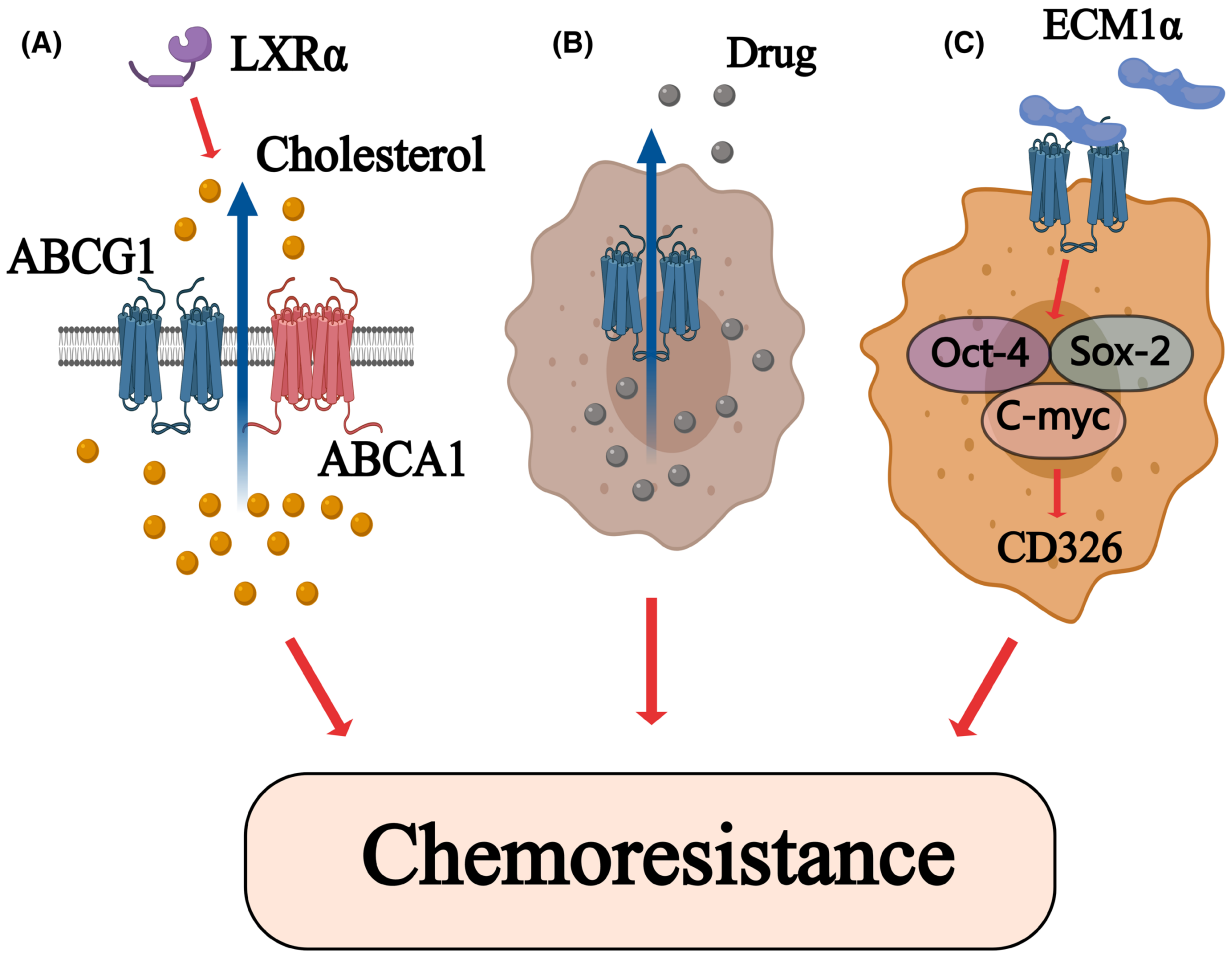
(A) The LXRα‐induced cholesterol efflux through ABCG1 and ABCA1 mediates cancer cell chemoresistance.^41^ (B) The upregulated expression of ABCG1 in rapidly growing cancer cells triggers an independent drug efflux mechanism to induce chemoresistance.^13^, ^39^ (C) Expression of ABCG1 in CD326+ ovarian cancer cells activates the expression of stem cell transcription factors to enrich the population of CD326+ cells and to confer cisplatin chemoresistance to them.^26^

The detailed mechanisms in chemoresistance induced by ABCG1 is still not very clear, but this may be associated with the level of cholesterol‐induced cancer cell viability. The decreased expression of ABCG1 and ABCA1 induced by LXRs inhibition enhances the chemosensitivity of adrenocortical carcinoma cells to mitotane.^41^ (Figure 3) In addition to cholesterol efflux, ABCG1 may induce chemoresistance and improve cancer cell viability by an independent efflux mechanism, which may be more related to ABCG1 expression in nucleus rather than cytoplasm.^13^, ^39^

Apart from the efflux role, ABCG1 is also confirmed to participate in several signaling pathways to enhance cancer cell chemoresistance. The activation of Wnt/β‐catenin signaling is detected to induce oxaliplatin resistance in HCC.^42^ In this pathway, ABCG1 acts as a downstream protein and significantly induces chemoresistance to improve cell survival. In ovarian cancer, the upregulated ABCG1 increases the expression of stem cell transcription factors to enhance the CD326‐mediated stemness, conferring cancer cell cisplatin resistance^26^ (Figure 3).

## 6 ABCG1 CONFERS STEMNESS TO TUMOR CELLS

Numerous reports indicate that ABCG1 promotes cancer cell stemness and tumor growth. The correlation between ABC transporters and tumor stem cells has been reported a lot. Among ABC transporters, ABCG2 and ABCB1 are well‐studied. Both of them are critical in maintaining undifferentiated state of cells.^59^, ^60^ Actually, the expression of ABCG1 is also associated with cancer stem cells (CSCs). Consistent with a tumor‐promoting role, ABCG1 enhances cell stemness properties and confers CSCs a survival advantage to induce tumor formation, metastasis, relapse, and resistance toward treatment in multiple cancers.^10^, ^30^, ^35^, ^61^

### 6.1 ABCG1/HIF‐1Α/PDK1 signaling axis confers tumor stemness

ABCG1 is indicated to confer tumor stemness through activating the H19/HIF‐1α/PDK1 signaling pathway to induce rapid cancer cell growth and adaption to the condition of hypoxia and insufficient energy supply. According to Otto Warburg, cancer cells prefer the energy and intermediates produced by aerobic glycolysis even in the presence of oxygen to sustain their rapid cell growth and active proliferation.^62^ This reliance on glycolysis can be further accentuated under the hypoxic conditions.^52^ Glycolysis promotes the abilities of cancer cell self‐renew and stem‐like property maintenance.^30^, ^63^, ^64^ The potential regulatory mechanisms are associated with the H19/HIF‐1α/PDK1 signaling axis.^30^ Under rapid growth and aggregation of cancer cells, hypoxia and necrosis appear in the central area of tumor mass. Subsequently, long non‐coding RNA (lncRNA) H19 and Hypoxia‐inducible factor 1‐α (HIF‐1α) are both induced to adapt the changed environment. Besides, H19 interacts with miRNAs in the cytoplasm and functions as an endogenous sponge for miRNAs, which in turn leads to the increased HIF‐1α expression.^30^ A report showed that Pyruvate dehydrogenase kinase 1 (PDK1) is a direct target of HIF‐1a.^44^ The accumulation of PDK in hypoxic regions may activate glycolysis and promote cell growth and proliferation. Namba et al. reported that ABCG1 acts as an upstream regulator of HIF‐1a and targeting of ABCG1 reduces HIF‐1a levels in tumor, resulting in cell death.^32^ Thus, ABCG1 may confer cell stemness and maintain stem‐like properties by enhancing aerobic glycolysis of malignant tumors through the HIF‐1a/PDK1 signaling axis.

### 6.2 ABCG1 enhances survival of CSCs through suppressing ER stress

In addition to signaling pathway, ABCG1 is also detected to support the growth of cancer stem cells through suppressing ER stress. The disruption of intracellular hemostasis may result in accumulation of unfolded or misfolded proteins, causing ER stress.^65^ The response to ER stress can promote cellular repair and cell viability through a series of adaptive mechanisms. But when ER stress is overwhelming, cells go through apoptosis.^66^ Recently, Chen et al. reported that ABCG1 reduces apoptosis of glioma stem cells (GSCs) through suppression of ER stress. Knockdown of ABCG1 increases and activates BiP and CHOP to break ER hemostasis to promote apoptosis in glioma.^34^, ^35^ In agreement with an active role in tumor development, ABCG1 is confirmed to promote glioma cell growth via regulation of ER stress. However, more detailed interactions between ABCG1 and ER stress in other tumors need further investigations.

## 7 ABCG1 IN TUMOR IMMUNAL MICROENVIRONMENT

Apart from tumor cells, ABCG1 also functions actively in tumor microenvironment to influence tumor initiation and progression. The interaction between tumor cells and microenvironment is not a new topic. Indeed, the TME is reported to play either anti‐tumorigenic or pro‐tumorigenic role in tumorigenesis depending on different stages of cancer development and different organs.^67^ The TME consists of various non‐cancerous cells including fibroblasts, endothelial cells, and immune cells together with non‐cellular components including the extracellular matrix (ECM) and substances secreted by cells like chemokines, cytokines, and growth factors.^68^

Among those components, immune cells are detected to actively engage in tumor formation and tumor growth.^69^ In response to tumor‐derived signals, most antitumor functions are downregulated, and immune cells in TME not only fail to exert antitumor effects, but also facilitate tumor growth.^70^ Macrophages, one of the innate immune cells that link inflammation and cancer, can be classified into two subtypes including M1 acting as tumor‐fighters and M2 acting as tumor‐promoters.^71^, ^72^ M2 cells promote tumor progression, and high levels of TAMs (tumor‐associated macrophages, with more M2 phenotype) are often, although not always, correlated with a poor prognosis.^73^ In addition, TAMs can affect adaptive immune response through recruiting T regulatory cells (Tregs.) to inhibit tumor‐fighting cells.^74^

**FIGURE 4:**
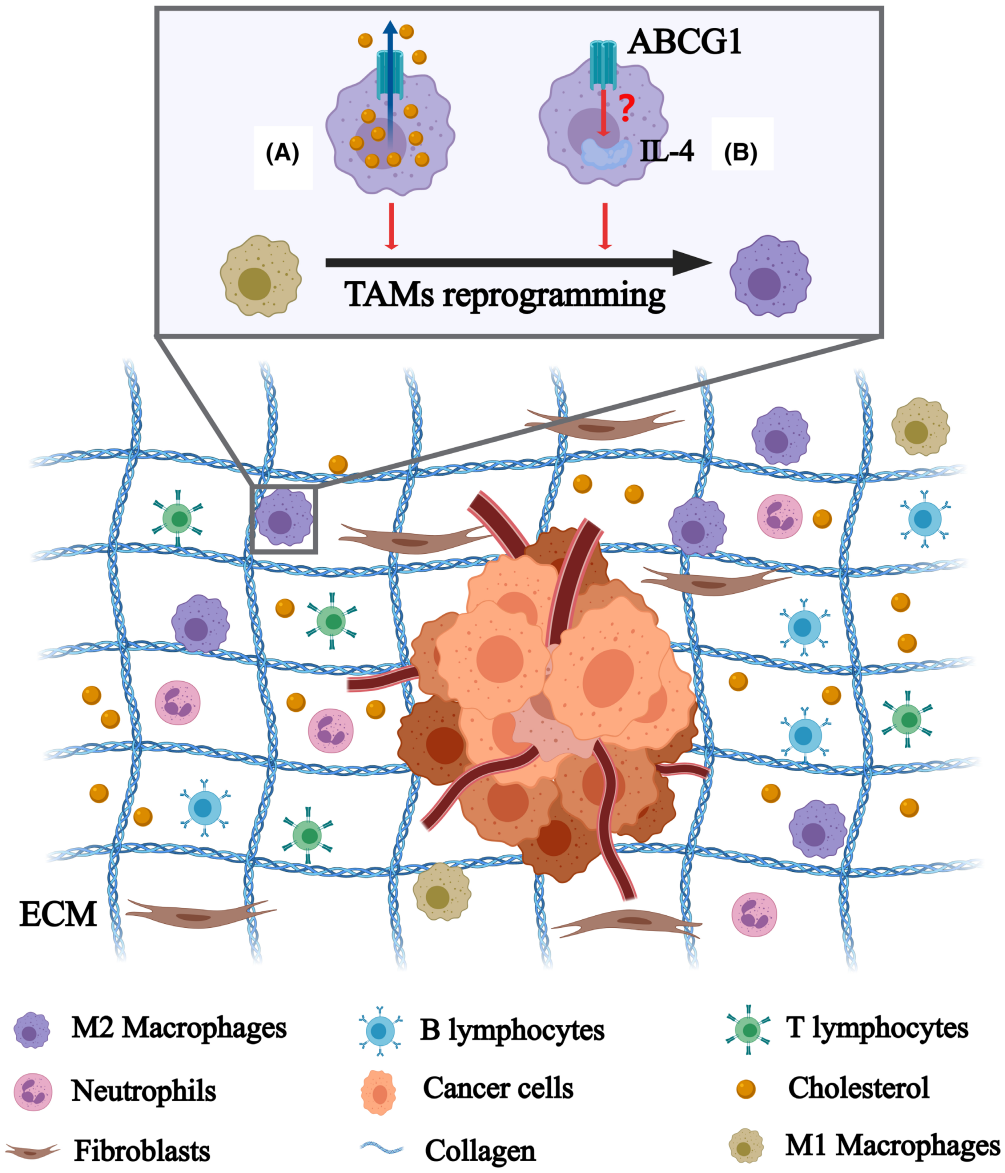
Macrophages play a vital role in tumor progression among all components within TME. (A) ABCG1 induces the polarization of macrophages from M1 to M2 to promote tumorigenesis through mediating intracellular cholesterol efflux.^77^ (B) ABCG1 may regulate the reprogramming of TAM‐like macrophages mediated by IL‐4 to promote tumorigenesis.^28^, ^31^

ABCG1 is known to promote reverse cholesterol transport in macrophages, showing an anti‐inflammatory and antiapoptotic effect with HDL through decreasing the expression of inflammatory cytokines and chemokines secreted from macrophages.^75^ The loss of ABCG1 is demonstrated to enhance the expression of proapoptotic genes inducing macrophage apoptosis.^76^ In tumor, the absence of ABCG1 inhibits cancer cell growth through modulation of macrophage function as a mediator of tumor immunity. ABCG1 may induce the phenotype shift of macrophages from M1 to M2 through efflux of cholesterol or 7‐KC, contributing to tumor progression and prolonged cancer cell survival^77^ (Figure 4). The balance between tumor‐promoting and tumor‐fighting immune cells within the TME is broken by aberrant expression of ABCG1, leading to a tumor‐favorable environment. Besides, a report recently showed that ovarian cancer cells promote membrane cholesterol efflux from macrophages through ABC transporters to induce IL‐4‐mediated reprogramming of TAMs to drive tumor progression.^28^ Although which of ABC transporters plays the key function remains unclear, ABCG1 is still a potential research subject, because it attenuates cholesterol levels in TAM‐like macrophages to induce tumor‐promoting actions in human lung cancer cells.^31^ Above all, ABCG1 may function actively in tumor microenvironment especially in macrophages through the regulation of cholesterol hemostasis in cells. Thus, inhibition of cholesterol efflux transporters like ABCG1 represents a potential strategy to reprogram TAMs for suppression of tumor growth.

## 8 RELEVANT REGULATION OF TUMORS BY ABCG1

In tumor progression, the expression of ABCG1 interacts with non‐coding RNA and some other signal molecules. For instance, Circ_ASPH, is reported to attenuate the suppression of ABCG1, resulting in the upregulation of ABCG1 to promote cancer progression^36^ (Table 2). Overexpression of miR‐129‐5p inhibits the expression of ABCG1 and other ABC transporters to reduce drug resistance in gastric cancer.^78^ HOXB13 induces metastasis and cisplatin resistance through activating the ABCG1/EZH2/Slug signaling.^37^ In lung cancer, the progenitor AT2 cells express high ABCG1 levels through the NF‐kB/ECM1/α6β4 axis to confer self‐renew and tumor initiation capacities, suggesting ABCG1 may be a potential biomarker for early diagnosis.^16^ In addition, ABCG1 may be a potential target binding to some molecules of signal pathways including Hedgehog‐GLI, and the increased ABCG1 is linked to the enhanced chemoresistance in cancer cells.^33^ In ovarian cancer cells, ABCG1 is reported to enhance tumor formation and progression by inducing the expression of C‐C motif chemokine ligand 20 (CCL20).^61^ Above all, ABCG1 functions in diverse signal modulation actions in different cancers, and usually acts as a pro‐tumorigenic role. A comprehensive understanding of ABCG1 may unveil a promising strategy for effective treatment of multiple cancers.

**TABLE 2:** Relevant molecules that regulate the expression of ABCG1 in multiple cancers and subsequent effects on tumor progression.

Subject	Cancer type	Expression	Effect	References
miR‐518	Cholangiocarcinoma	Down	Inhibits tumor progression	[36]
miR‐129‐5p	Gastric cancer	Down	Reduces drug resistance	[78]
Circ_ASPH	Cholangiocarcinoma	Up	Attenuates the suppression of miR‐518	[36]
HOXB13	Lung adenocarcinoma	Up	Induces metastasis and cisplatin resistance	[37]
NF‐κB/ECM1/α6β4 axis	Lung cancer	Up	Tumor initiation	[16]
Hedgehog‐GLI	Colorectal cancer	Up	Induces chemoresistance	[33]
CCL20	Ovarian cancer	Up	Tumor formation and progression	[61]

## 9 CONCLUSION AND PERSPECTIVES

In the last few decades, ABCG1 has been widely studied in aspects of cholesterol homeostasis and atherosclerosis while the function in tumor is relatively less described. Actually, ABCG1 is confirmed to be associated with various tumors and be involved in many aspects of tumor progression. The effects of ABCG1 on tumor development can be either anti‐cancerous through LXR induced cholesterol efflux, or tumor‐promoting via multiple proliferation, chemoresistance, and stemness‐related molecular signals and interaction with tumor microenvironment. The specific role is tumor‐dependent and results from multifactorial interactions. Abnormal expression of ABCG1 in rapid growing cancer cells means ABCG1 may be the key factor for tumor progression, which also provides a new target for drug therapy.

As the underlying causes of various cancers are gradually identified, precision medicine therapies are inevitably becoming the focus in drug research in order to reduce the general cytotoxicity and severe side effects.^79^ Therapies targeting cancer cells can be divided into two prime methods: pathway‐based therapy and immunotherapy.^79^ The targeted approaches aim to inhibit molecules that are vital to cancer progression and metastasis yet facing the challenge of acquired drug resistance, whereas immunotherapy to generate a sustained response against cancer cells through modulating immune systems yet the adaptability and evolution of tumor cells can still lead to an impaired immune response.^80^ As mentioned above, ABCG1 participates in cancer growth and maintenance via several signaling pathway by interacting with immune cells within tumor microenvironment. The ABCG1‐targeted therapy may combine the benefits of targeted therapy and immunotherapy, shedding a light on the high ABCG1‐expression patients with poor prognosis. Unfortunately, there is little research on ABCG1‐mediated cancer drugs at present.

Despite the promising clinical application of ABCG1, a few questions are still remained unanswered. Since the ABCG1‐mediated cholesterol efflux is involved in both cell death through depriving necessary lipids and cell growth through decreasing accumulation of toxic lipids, what factors regulate the balance between the two outcomes? Under what circumstances can we control the status of cells via ABCG1 regulation? It is mentioned that ABCG1 may act like a kinase, what is the exact mechanism of downstream signaling on cancer cell growth? Since the kinase role of ABCG1 is pointed in ovarian cancer, whether it has the same function in other types of tumors? Is the correlation between ABCG1 and ER stress also exists in other tumors? whether ABCG1 acts pivotally in the IL‐4‐mediated reprogramming of TAM in tumor microenvironment or not? Obviously, further researches are largely needed to answer more details.

## AUTHOR CONTRIBUTIONS

**Xu Xinyi:** Investigation (equal); writing – original draft (lead); writing – review and editing (equal). **Yang Gong:** Conceptualization (lead); investigation (equal); writing – review and editing (equal).

## FUNDING INFORMATION

This study was supported by the grants#81572553/81772789/81372797 for G.Y.

## CONFLICT OF INTEREST STATEMENT

The authors declare no potential conflicts of interest.

## ETHICS STATEMENT

Not applicable.

## Data Availability

Data sharing is not applicable to this article as no new data were created or analyzed in this study.
